# Impact of hydrogen peroxide photolysis on viable bacterial count and composition of in vivo dental biofilm—an ex vivo study

**DOI:** 10.1186/s12903-025-07588-6

**Published:** 2025-12-30

**Authors:** Midori Shirato, Anna Lehrkinder, Keisuke Nakamura, Taro Kanno, Peter Lingström, Ulf Örtengren

**Affiliations:** 1 Department of Cariology, Institute of Odontology, Sahlgrenska Academy, University of Gothenburg, Gothenburg, SE 405 30 Sweden; 2 Department of Advanced Free Radical Science, Tohoku University Graduate School of Dentistry, 4-1 Seiryo, Aoba-ku, Sendai, 980 8575 Japan; 3 Department of Material Science and Technology, Faculty of Odontology, Malmö University, Malmö, Malmö, SE 214 21 Sweden

**Keywords:** Biofilms, Dental caries, Caries treatment, Hydrogen peroxide

## Abstract

**Background:**

A bactericidal technique utilizing hydrogen peroxide (H₂O₂) photolysis, which generates hydroxyl radicals, was developed. Since it has demonstrated the potential to kill *in vitro Streptococcus mutans* biofilms, this technique has the possibility to be applied to dental caries. However, its efficacy on in vivo cariogenic dental biofilms remains unclear. This study aimed to evaluate the bactericidal effect of H₂O₂ photolysis on in vivo biofilms obtained from volunteers, focusing on its potential application in dental caries treatment.

**Methods:**

Sixteen participants, prescreened for the presence of *S. mutans* and/or lactobacilli in their saliva, wore custom-made splints with hydroxyapatite (HA) discs for 5 days. The discs with 5-day-old biofilms were subjected to H₂O₂ photolysis or control treatments such as H₂O₂, light-emitting diode (LED) irradiation, or water for 90 s, followed by bacterial culturing and quantitative real-time polymerase chain reaction (qPCR). Statistical significances were assessed using the Steel–Dwass test. Scanning electron microscopy (SEM) and 16S rRNA gene sequencing were also used for biofilm evaluation. Discs with treated-biofilms were reinserted for an additional 5 days to evaluate biofilm regrowth and community shifts.

**Results:**

SEM confirmed biofilm formation on HA surfaces. Bactericidal assays showed that the 90-s treatment with H₂O₂ photolysis significantly reduced viable bacterial counts, achieving a 3.5 log colony-forming unit (CFU)/specimen for total bacteria and a 2.6 log CFU/specimen for total streptococci, compared with 5.9- and 5.4 log CFU/specimen in the untreated controls, respectively (*p* < 0.05). qPCR confirmed that the bacterial proportion was initially equivalent across the groups and remained unchanged after regrowth. The 16 S sequencing revealed a diverse microbial community dominated by *Streptococcus spp*. No significant differences in alpha or beta diversity were observed between the treatment groups even after regrowth.

**Conclusion:**

These findings suggest that the H₂O₂ photolysis technique can kill bacteria within in vivo biofilms. The observed bactericidal effect supports the potentiality of H₂O₂ photolysis as a promising adjunctive approach for dental caries.

## Background

Maturation of dental biofilms can cause dysbiosis, leading to a favorable environment for the induction of dental caries and other oral diseases [1, 2]. The dysbiosis of supragingival biofilms is strongly associated with caries development. Host factors, such as frequent sugar consumption, poor oral hygiene, and reduced saliva flow, can induce dysbiosis and accelerate the activity of acidogenic and acid-tolerant bacteria in supragingival biofilms [3]. This in turn leads to environmental acidification and may result in tooth demineralization. *Streptococcus mutans* plays a crucial role in the initiation of dysbiosis via sucrose metabolism. Additionally, other oral bacteria known as early acidogenic or aciduric colonizers, such as non-mutans streptococci, *Actinomyces spp*., *Lactobacillus spp*., and Bifidobacteria, engage in polymicrobial interactions with *S. mutans*, further promoting biofilm acidification [3–5]. Therefore, effective management of dental biofilms is crucial for the prevention and treatment of dental caries. However, the structural complexity and protective environment of dental biofilms present significant challenges for their appropriate management. Dental biofilms comprise a diverse bacterial community embedded in an extracellular polymeric substance (EPS) [5], which enhances resistance to antimicrobial agents (such as antiseptics) compared with planktonic bacteria [6]. Furthermore, the complex anatomical structure of teeth, where dental biofilm is often formed, adds to the difficulty of thorough removal during dental treatments [7, 8]. Considering these challenges, although several adjunctive methods have been introduced to complement mechanical dental biofilm removal and manage infected tooth surfaces, a consistently effective approach has not yet been established.

Hydrogen peroxide (H_2_O_2_) photolysis has emerged as a promising adjunct to antimicrobial treatment in dentistry [9–11]. H_2_O_2_ photolysis is a photochemical reaction that occurs when H_2_O_2_ is irradiated by light with wavelengths near or below 400 nm, generating hydroxyl radicals [12]. The hydroxyl radical is one of the reactive oxygen species and is known for its potent oxidative power [13, 14]. Owing to this property, hydroxyl radicals are expected to effectively kill bacteria by oxidizing their cellular components [15]. Because oral diseases such as dental caries are strongly related to bacteria, the application of H_2_O_2_ photolysis has great potential as a new adjunctive antimicrobial technique.

Previous in vitro studies have demonstrated that the H_2_O_2_ photolysis technique exerts strong bactericidal effects on various oral bacteria and biofilms [9–11]. Animal studies have demonstrated that applying H_2_O_2_ photolysis treatment to the oral mucosa and teeth does not provoke inflammatory cell infiltration in either the mucosa or dental pulp [16, 17]. Based on these findings, a clinical device utilizing H₂O₂ photolysis for periodontal treatment has been developed and proven clinically effective [18]. Beyond the periodontal treatment, its potential applications in caries treatment, endodontic therapy, and treatment of peri-implant diseases have been explored.

Several studies have provided evidence supporting this technology for dental caries treatment. Nakamura et al. reported the efficacy of H_2_O_2_ photolysis against *in vitro S. mutans* biofilm [19], whereas Shirato et al. demonstrated that it was more effective against *S. mutans* biofilms than conventional antiseptics, commonly used in the oral cavity, and antimicrobial photodynamic therapy (aPDT) [20], a method that also uses reactive oxygen species to kill bacteria [10]. Both studies used hydroxyapatite (HA) discs containing biofilms to mimic enamel surfaces. Additionally, optimal light wavelengths have also been investigated for the application, with shorter wavelengths (particularly 365 nm) showing greater bactericidal activity [21]. Although these in vitro studies highlighted the potential of the H_2_O_2_ photolysis technique for caries treatment, its efficacy against supragingival biofilms remains poorly understood.

Based on these studies, we hypothesized that the hydroxyl radicals generated by H_2_O_2_ photolysis would retain their bactericidal potential even when applied to human in vivo biofilms corresponding to supragingival biofilms. Studies have employed dental splints with discs to collect human dental biofilms and have successfully analyzed their microbial composition [22, 23]. This method of using dental splints would be a promising technique for obtaining in vivo biofilm to test this hypothesis. Additionally, as the methodology of using biofilms formed on HA discs has been standardized and well established [10, 19], experiments using in vivo biofilms formed on HA discs (i.e., ex vivo experiments) can serve as a bridge between previous in vitro findings and future in vivo applications. Thus, this study aimed to evaluate the bactericidal effect of H_2_O_2_ photolysis on in vivo dental biofilms, with a particular focus on cariogenic biofilms, obtained from volunteers using custom-made splints.

## Methods

### Enrolment and informed consent

The study protocol was reviewed and approved by the Swedish Ethical Review Authority (Etikprövningsmyndigheten; Dnr 2021–03933). Healthy adults were recruited and underwent a screening examination. The inclusion criteria were: (i) age > 20 years, (ii) presence of at least 24 permanent teeth, and (iii) presence of cariogenic bacteria (*S. mutans* > 100,000 colony-forming unit (CFU)/ml and/or lactobacilli > 10,000 CFU/ml). Stimulated saliva collected from the candidates was used for bacterial counting by culturing on mitis-salivarius agar (Becton, Dickinson and Company, Franklin Lakes, NJ, USA) containing sucrose and bacitracin (MSB) for *S. mutans* and Rogosa agar (Becton, Dickinson and Company) for lactobacilli. The cutoff value for bacterial count was set at the level generally considered to indicate an increased or high caries risk [24–26]. The candidates chewed paraffin gum until approximately > 2 mL of saliva was produced. The collected saliva was immediately transferred into the medium (VMG IIS) [27] and stored in a refrigerator until culture. The exclusion criteria were: (i) presence of systemic disease, (ii) smoking or snuff use, (iii) use of antibiotics and probiotics within 30 days prior to the study, (iv) ongoing orthodontic treatment, and (v) pregnancy. At the screening, the investigator provided written and verbal information about the study, and the informed consent form was signed by all the participants.

### Sample size calculation

The sample size was calculated using JMP Pro software (version 17.0; SAS Institute, Cary, NC, USA). The primary outcome was defined as the CFU per specimen obtained immediately after treatment (total viable bacterial counts). The expected mean values and standard deviations (SD) were based on the findings from our previous in vitro biofilm study [10]. To achieve 80% statistical power with a significance level of 5%, a minimum of 13.25 discs per treatment group (total 53 discs) was calculated. Seventeen discs per group (68 discs in total) was planned to compensate for possible dropouts. One disc per treatment group (i.e., four discs in total) from each participant had to be subjected to the determination of CFUs immediately after treatment; therefore, 17 participants who met the inclusion criteria were planned to be recruited.

### Hydroxyapatite discs

HA discs with a diameter of 4.5 mm and thickness of 2.0 ± 0.05 mm were fabricated to simulate the enamel surfaces. HA powder (HAP-200, Taihei Chemical, Osaka, Japan) was placed in a cylindrical metal mold with a diameter of 6 mm and uniaxially pressed at 20 MPa using a Mini Lab Press (Labonect, Osaka, Japan), followed by cold isostatic pressing at 200 MPa using the Labo Press (Labonect). The resulting powder compacts were sintered at 1250 °C (heating rate: 10 °C/min, holding time: 2 h, and cooling rate: 4 °C/min). The sintering conditions were determined based on the results of preliminary experiments conducted with reference to a previous study [28]. The sintered HA discs were polished using #1500 grit (9 μm) silicon carbide paper (Trusco Nakayama, Tokyo, Japan). The specimens were characterized by using an optical interferometer for surface roughness, Vickers hardness tester, X-ray diffraction for crystallin structure, and Archimedes method for relative density. The average height distribution (S_a_) was 0.09 (SD: 0.02), Vickers hardness was 470.1 (SD: 15.9) Hv, crystallin structure was hexagonal phase of HA, and relative density was 97.7%. The discs were then autoclaved at 121 °C for 20 min before being attached to the splint.

### Experimental splints

**Fig. 1:**
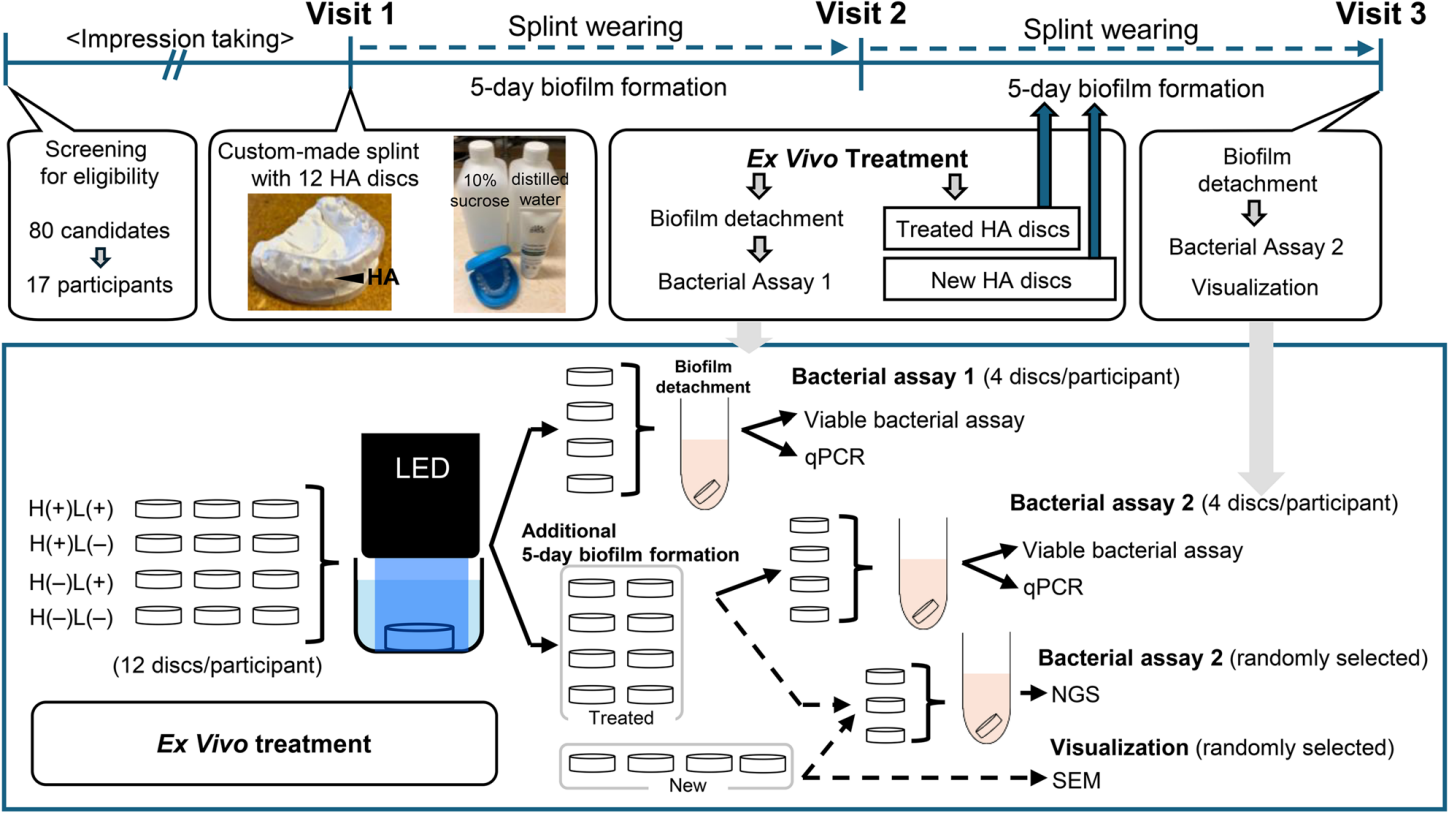
Study design. Eighty candidates are screened for eligibility. Custom-made splints are fabricated from dental impressions before Visit 1. **Visit 1:** Participants receive splints with 12 hydroxyapatite discs, 10% sucrose solution, distilled water, and non-fluoride toothpaste and are instructed to wear the splints for 5 days. **Visit 2:** Participants returned with the splints, allowing the discs to undergo ex vivo treatment (three discs/treatment). All 12 discs are removed from the splint for bactericidal assay. Four treated specimens are used for viable bacterial assay and qPCR analysis, referred to as “Bacterial Assay 1.” The remaining eight treated specimens and four new intact discs are reattached to the split. The participants wore the splint for five additional days. **Visit 3:** Participants returned the splints. The four treated specimens (10-day-old biofilm) are removed for viable bacterial assay, qPCR analysis, and another four treated specimens and new discs (5-day-old biofilm) are randomly subjected to next-generation sequencing (NGS), referred to as “Bacterial Assay 2.” Scanning electron microscopy (SEM) is randomly performed on new discs for “Characterization”. H_2_O_2_ photolysis is referred to as H(+)L(+), H_2_O_2_ alone as H(+)L(-), water with LED irradiation as H(-)L(+), and water alone as H(-)L(-), respectively

Custom-made mandibular splints were fabricated at the TIC DP Göteborg Dental Laboratory (Gothenburg, Sweden), using impressions from each participant. A thermoplastic material (ERKOFLEX 120 mm × 5.0 mm; Erkodent, Eric Kopp GmbH, Pfalzgrafenweiler, Germany) was pressed onto the mandibular dentition model and trimmed, as shown in Fig. 1. Twelve HA discs (six on each side) were pressed onto the buccal surface of the splint, where spaces for removable HA discs were formed using the Hot Air Burner (Erkodent). Autoclaved HA discs were mounted onto the designated spaces on the splint immediately before being handed to each participant.

### Instructions for the participants

At Visit 1 (Fig. 1), each participant received a splint with 12 HA discs, distilled water, 10% sucrose solution, and non-fluoride toothpaste. They were instructed to wear the splint for as long as possible over a period of 10 days and store it in distilled water when not in use. To promote cariogenic biofilm formation, the participants were instructed to dip the splint in the 10% sucrose solution for 1 min three times daily [29, 30]. Additionally, they were instructed to brush their teeth with the provided non-fluoride toothpaste only and to avoid touching the disc surfaces and to remove the splint when eating, brushing their teeth, or drinking anything other than water. During the study period, the participants were scheduled for two visits, at 5 days (Visit 2) and 10 days (Visit 3) after Visit 1. 

### Ex vivo treatment of H_2_O_2_ photolysis

After 5 days of wearing the splint (Visit 2, Fig. 1), all the 12 HA discs with biofilms were detached for ex vivo treatment. The specimens were divided into four groups (*n* = 3 for each group/participant), which were treated with H_2_O_2_ in combination with light-emitting diode (LED) irradiation (H(+)L(+), test group: H_2_O_2_ photolysis), H_2_O_2_ alone (H(+)L(−), positive control 1), water in combination with LED irradiation (H(−)L(+), positive control 2), or water alone (H(−)L(−), negative control). A 3% H_2_O_2_ was prepared by diluting 30% H_2_O_2_ (Sigma-Aldrich, St. Louis, MO, USA) in sterile water. An LED spot-curing device (OmniCure LX500, Excelitas Technologies Corp., Mississauga, ON, Canada) with a head that emitted ultraviolet light at a wavelength of 365 nm was used as a light source. A lens with an irradiation area of 6 mm diameter at a working distance of 18 mm was attached to the LED head. LED power was adjusted to 283 mW using a power meter (FieldMate; Coherent, Santa Clara, CA, USA) to achieve an irradiance of 1000 mW/cm^2^. The specimen kept in saline until treatment was transferred to a 48-well plate containing 500 µl H_2_O_2_ for H(+) group or water for H(−) group. L(+) group was treated with LED irradiation for 90 s, while L(−) group was kept under a light shield for 90 s. After treatment, the specimens were rinsed twice with saline to eliminate the effect of H_2_O_2_ and then kept in saline. Experimental settings were optimized as in our previous studies [10, 19, 31]. One specimen from each treatment group (a total of four specimens) underwent a viable bacterial assay and quantitative real-time polymerase chain reaction (qPCR) as described below, whereas the remaining eight treated specimens were reattached to the splint along with four intact new HA discs. The participants continued wearing the splints for an additional 5 days to assess biofilm regrowth. At Visit 3, among the specimens reattached to the splint after treatment, one specimen from each treatment group was subjected to both viable bacterial assay and qPCR. The remaining and newly attached specimens at Visit 2 (5-day-old biofilm) were randomly subjected to next-generation sequencing (NGS) analysis. To visualize the representative 5-day-old biofilm, the newly attached specimens were also randomly subjected to scanning electron microscopy (SEM) (3 specimens in total). Additionally, the newly attached specimens were used to handle technical errors in the experiment at Visit 1, if necessary.

### Viable bacterial assay

Specimens from each treatment group (four in total) were placed in a microtube containing 300 µl of enzyme solution (1000 U/ml type I collagenase, 3.6 U/ml dispase; Gibco, Thermo Fisher Scientific, Waltham, MA, USA) and incubated at 37 °C for 2 h with vibration to detach the biofilms [32, 33]. The resulting suspensions were plated on blood agar (Microbiology Institute, Sahlgrenska Academy, Gothenburg, Sweden) to determine the total viable bacterial count. Selective media were also used to isolate specific bacteria: mitis-salivarius agar for total streptococci, MSB agar for *S. mutans*, and Rogosa agar for lactobacilli. The plates were anaerobically incubated at 37 °C in an anaerobic chamber controlled by a gas infuser (Coy Laboratory Products, Inc. Grass Lake, MI, USA) for blood agar and a simple anaerobic box for others for 48 h. After incubation, visible bacterial colonies were counted to determine viable bacterial counts. After the additional 5 days of wearing (Visit 3, Fig. 1), the biofilms on the treated specimens were collected and cultured as described above. Biofilm regrowth between the treatment groups was compared based on colony counts. Statistical analysis was performed to detect significant differences in log-CFU between the groups using JMP Pro 17.0. Data normality and variance homogeneity were assessed using the Shapiro–Wilk and Levene’s tests. Because the data were not normally distributed, the nonparametric Steel–Dwass test was used for group comparisons (*p* < 0.05).

### Quantitative real-time polymerase chain reaction

Both the 5-day-old biofilms subjected to the treatments and 10-day-old biofilms were analyzed using qPCR to identify *S. mutans*, total streptococci, total lactobacilli, and total bacteria. DNA was extracted from the same biofilm suspensions used for viable bacterial assay by a PureLink™ Microbiome DNA Purification Kit (Invitrogen™; Thermo Fisher Scientific), according to the manufacturer’s instructions. qPCR was performed on an MIC analyzer (Bio Molecular Systems; Upper Coomera, QLD, Australia) using the primers listed in Table 1 [34–36]. Each 20 µl reaction mixture contained 1x qPCRBIO Sygreen mix (PCR Bio-Systems, London, UK), 400 nM of each forward and reverse primers, and 2.5 µl of DNA template. All the amplifications were performed in duplicate, and the obtained data were analyzed using MIC software (Bio Molecular Systems). The data were statistically analyzed in the same way as the log-CFU using the Steel–Dwass multiple comparison test (*p* < 0.05).

**Table 1:** List of primers

	Primer’s sequence 5’-3’	References
*Streptococcus mutans*	Forward: CTACACTTTCGGGTGGCTTG	Choi et al. [36]
	Reverse: GAAGCTTTTCACCATTAGAAGCTG	
Total streptococci	Forward: YGTGCAATTTTTGGATAAT	Täpp et al. [34]
	Reverse: TTCTATAAGCCATGTTTTGT	
Total Lactobacilli	Forward: TGGAAACAGRTGCTAATACCG	Byun et al. [35]
	Reverse: GTCCATTGTGGAAGATTCCC	
Total bacteria (universal)	Forward: TGGAGCATGTGGTTTAATTCGA	Choi et al. [36]
	Reverse: TGCGGGACTTAACCCAACA	

### Next-generation sequencing for microbiome analysis

The 16S rRNA based metagenomic analysis was performed on 15 biofilm samples to investigate their microbial composition. To obtain an overview of the biofilms in this study, samples of 5-day-old intact biofilm and regrown biofilms treated with H(+)L(+) and H(−)L(−) were collected from five individuals randomly selected from the 17 participants (3 samples with different conditions per individual, 15 samples in total). DNA extraction was performed using GenElute™ Bacterial Genomic DNA Kit (Sigma-Aldrich). The library for the V3-V4 bacterial region of 16S rRNA was prepared using the Nextra XT DNA library preparation kit (Illumina Inc., San Diego, CA, USA), following the manufacturer’s instructions. Sequencing was performed using a MiSeq Reagent Kit V3 (300 cycles) and a MiSeq instrument (Illumina Inc.). Sequencing data were processed using a standardized workflow and the: nf-core/ampliseq pipeline [37, 38]. Briefly, the quality of the sequencing reads was checked using FastQC and adapters were trimmed using Cutadapt. Inference of amplicon sequence variants was performed using DADA2 [39], a software package in R, to correct sequences from amplicon errors. Taxonomic classification was also made by DADA2 with SILVA 138.1 prokaryotic SSU database as reference taxonomy. Downstream analyses such as alpha and beta diversity were plotted using QIIME2 [40], a bioinformatics platform, and phyloseq using R. DESeq2 and ANCOM/ANCOM-BC were used for significance testing.

### Visualization of 5-day-old biofilm by scanning electron microscopy

In vivo biofilms formed on the HA discs after 5 days of wearing the splint were visualized using SEM (Gemini SEM 450; ZEISS Microscopy, Oberkochen, Germany). Three samples from Visit 3 were randomly subjected to SEM. HA discs with the biofilms were fixed in Karnovsky’s solution (4% paraformaldehyde and 1% glutaraldehyde in 0.1 M sodium cacodylate buffer) for 30 min, rinsed five times, then transferred to 1% osmium, and incubated for 1 h at room temperature. After rinsing three times with water for 5 min, the discs were dehydrated in different concentration series of ethanol (30–100%) for 5 min in each. Finally, the discs were dried with hexamethyldisilazane and sputtered using a gold coater (Quorum Sputter Coater Q150T E; Quorum Technologies Ltd., Laughton, East Sussex, UK).

## Results

### Participants and biofilm sample characteristics

**Fig. 2:**
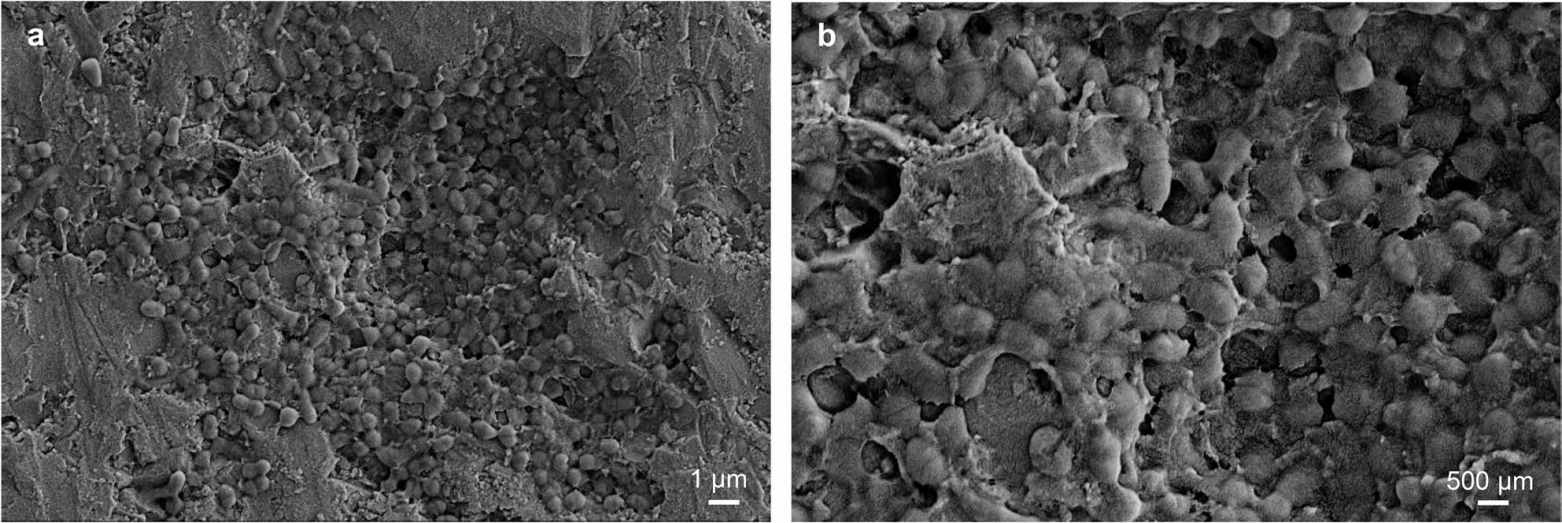
Representative scanning electron microscope (SEM) images of 5-day-old biofilm using this study. Representative SEM images of the biofilms are shown at (**a**) lower magnification and (**b**) higher magnification. The hydroxyapatite (HA) surfaces are covered with a biofilm. Streptococci-like bacteria enveloped in extracellular polymeric substances (EPS) are observed. Scale bars indicate (**a**) 1 μm and (**b**) 500 μm

Seventeen participants were initially enrolled from a pool of 80 candidates as planned. However, one participant withdrew due to illness before Visit 1. Consequently, a total of sixteen participants (four males and twelve females) completed the study. Average age was 25.8 (SD: 8.2) years, and the average number of teeth was 30.2 (SD: 2.2). The participants did not have any active carious lesions, although they had experienced caries earlier. Initially, they had an average of 5.5 log CFU/ml of both *S. mutans* and lactobacilli in their saliva. In vivo biofilms were developed on the HA discs by wearing a custom-made splint for a total of 10 days. Characterization of in vivo biofilms using SEM revealed that the HA disc surface was covered with streptococci-like bacteria after 5-day of wearing the splint (Fig. 2a). At a higher magnification of the SEM image, the bacterial cells were embedded in the EPS (Fig. 2b).

### Viable bacterial assay

**Fig. 3:**
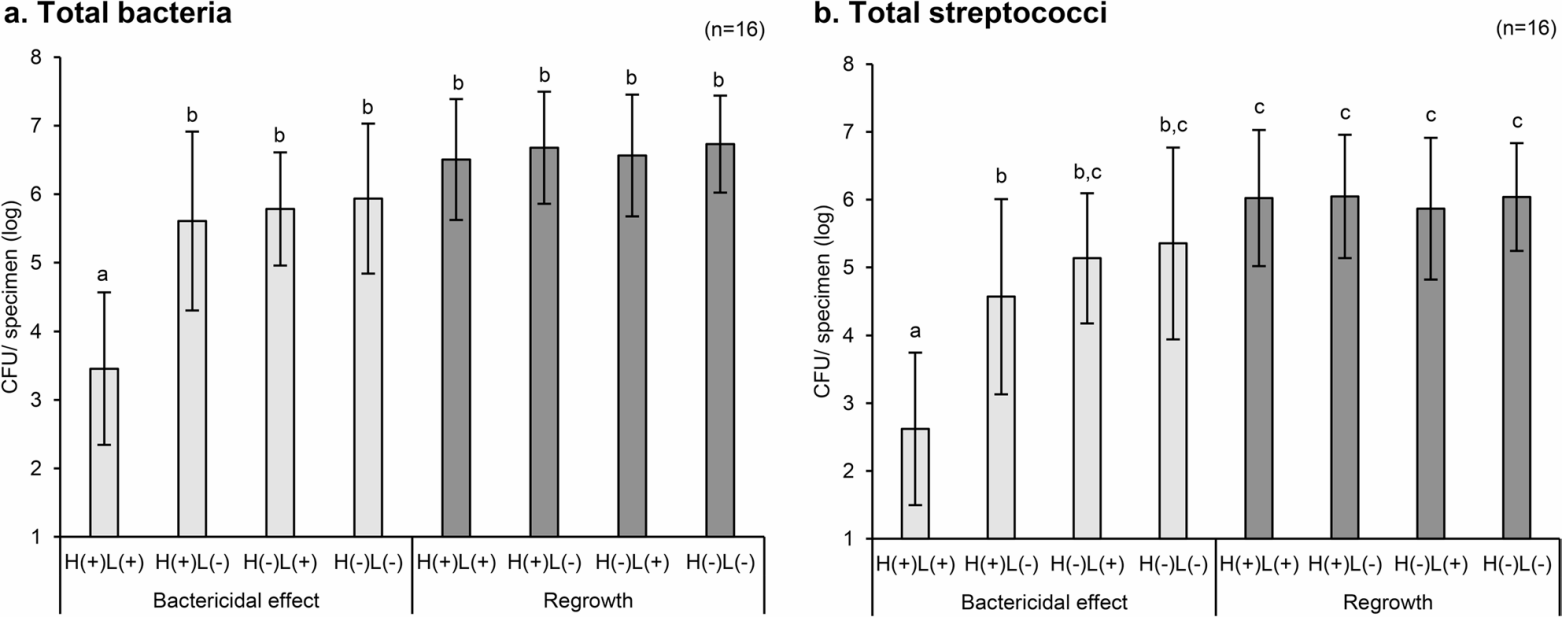
Evaluation of bactericidal effect and biofilm regrowth. Viable bacterial counts are determined for (**a**) total bacteria and (**b**) total streptococci in the biofilms. “Bactericidal effect” represents counts measured immediately after treatment with: H_2_O_2_ photolysis [H(+)L(+)], H_2_O_2_ without LED irradiation [H(+)L(-)], water with LED irradiation [H(-)L(+)], and water without LED irradiation [H(-)L(-)]. “Regrowth” represents counts measured after an additional 5 days of biofilm formation on treated discs. Values and error bars indicate the mean and standard deviation, respectively (*n* = 16 per group). Regardless of time point, all data are pooled and analyzed collectively. Different letters above the columns indicate significant differences (*p* < 0.05) between different groups

Treatment with H_2_O_2_ photolysis (H(+)L(+)) significantly reduced the number of bacteria in the biofilm compared with the other treatment groups (Fig. 3). Specifically, H(+)L(+) resulted in a 3.5 log CFU/specimen for total bacteria and a 2.6 log CFU/specimen for total streptococci, whereas the control group (H(−)L(−)) exhibited 5.9- and 5.4 log CFU/specimen, respectively. Treatment with H_2_O_2_ alone (H(+)L(−)) achieved an approximate 1 log reduction, with total streptococci showing slightly higher sensitivity to H_2_O_2_ than total bacteria. Exposure to LED irradiation alone (H(−)L(+)) resulted in less than 1 log reduction. In some samples, *S. mutans* and lactobacilli were not detected; specifically, *S. mutans* was detected in 6 out of 16 participants, and lactobacilli was detected in 7 out of 16 participants. Moreover, their CFU counts varied substantially among the specimens. Therefore, the results of total bacteria and total streptococci, which were detected in all samples, are shown in Fig. 3. According to the evaluation of biofilm regrowth, no significant differences in log-CFU were observed between the treatment groups after an additional 5-day of wearing the splint.

### qPCR analysis

**Fig. 4:**
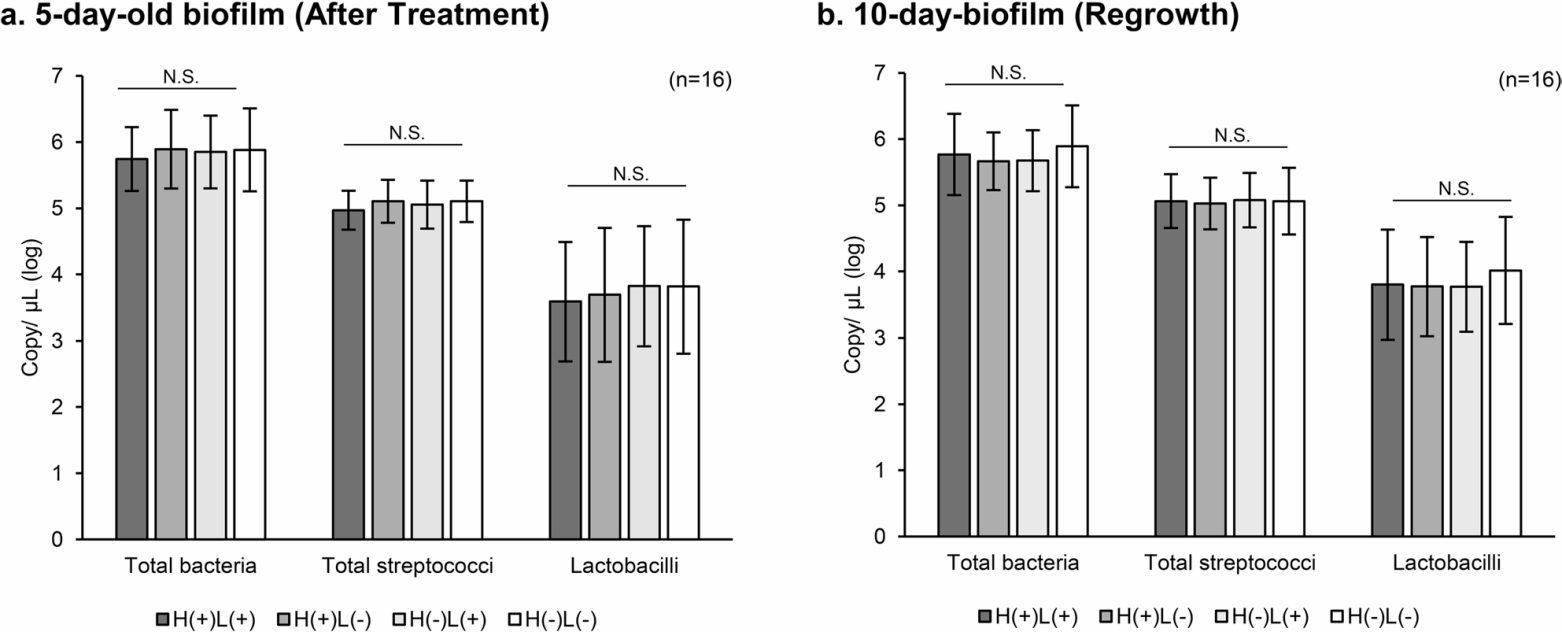
Bacterial proportion in the biofilms analyzed by qPCR. qPCR results of biofilms (**a**) after treatment (5-day-old biofilm) and (**b**) after regrowth (10-day-old biofilm). Total bacteria, total streptococci, and lactobacilli are shown separately for each treatment group, and treatment groups are statistically analyzed separately at each time point. Values and error bars indicate the mean and standard deviation, respectively (*n* = 16 per group). N.S.= not significant

Total streptococci and lactobacilli were detected in all samples from either 5- or 10-day-old biofilms. In contrast, *S. mutans* detection was inconsistent. For instance, in 5-day-old biofilm, *S. mutans* was detected in three samples from H(+)L(+) group, two samples from H(−)L(+) group, four samples from H(+)L(−) group, and eight samples from H(−)L(−), respectively (data not shown). With comparable data, excluding *S. mutans*, there were no significant differences in bacterial loads among the treatment groups for either the 5- or 10-day-old biofilms (Fig. 4). Additionally, the relative proportions of total bacteria, streptococci, and lactobacilli were similar across groups. As qPCR does not distinguish between live and dead cells, the results of the 5-day-old biofilms suggested that all treatment groups initially had comparable bacterial loads with similar bacterial categories. Consequently, the proportions observed before and after treatment were also comparable.

### Microbiome analysis by NGS

**Fig. 5:**
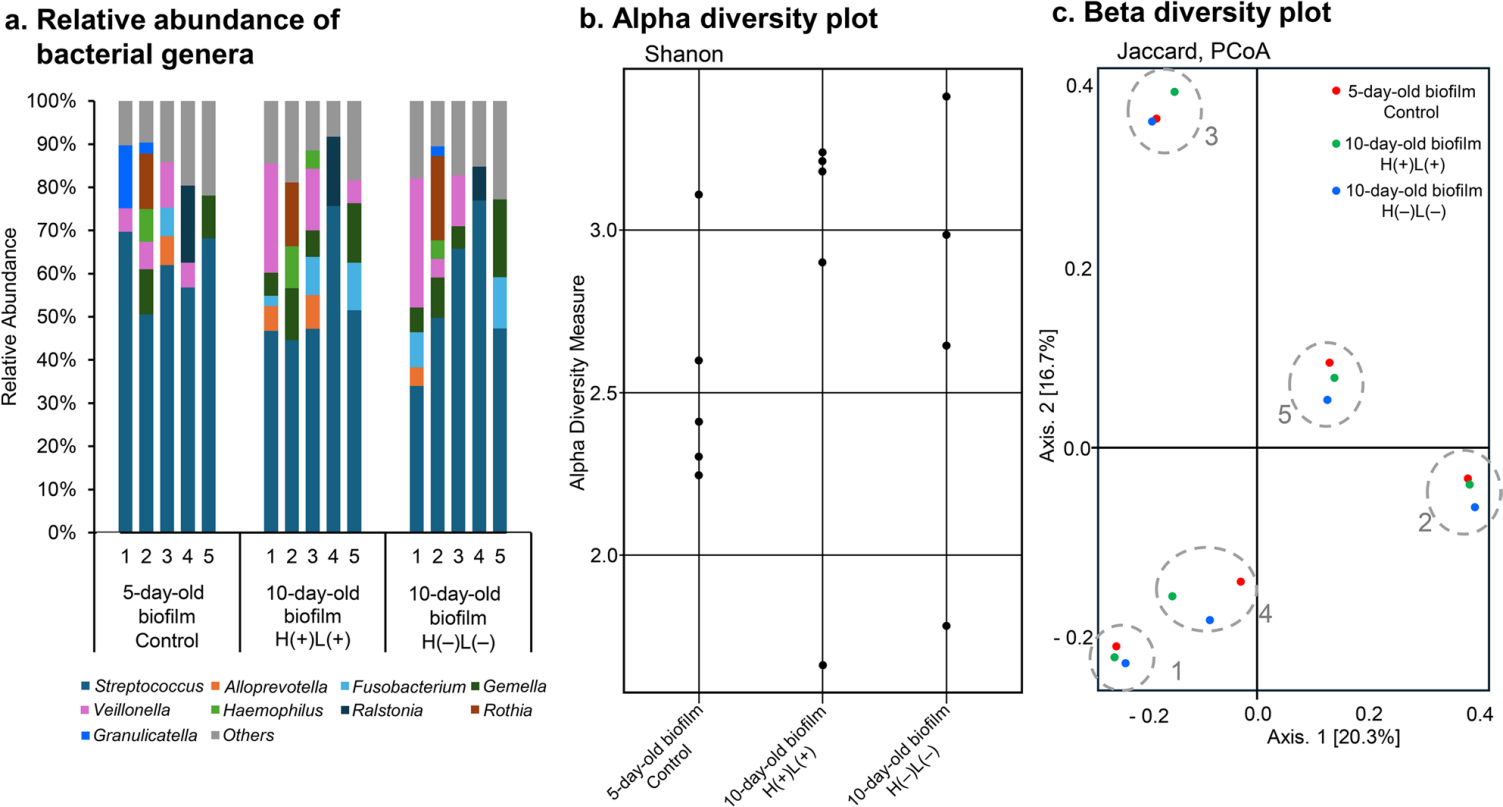
Microbiome analysis based on next-generation sequencing (NGS). The 16 S metagenomic analyses are performed on 15 biofilm samples derived from five individuals randomly selected from the study participants, namely subjects 1, 2, 3, 4 and 5. The samples include 5-day-old intact biofilm (Control), 10-day-old biofilm treated with H(+)L(+), and 10-day-old biofilm treated with H(−)L(−), with each sample collected per individual. **a** Relative abundance of bacterial genera. Under 2% detection genera represent “Others”. **b** Alpha diversity plot based on the Shannon diversity index. Individual sample values are shown separately at each condition. **c** Principal coordinates analysis (PCoA) plot of beta diversity based on Jaccard distance. Samples from the same individual at different conditions are clustered together, as indicated by each circle

Genus level analysis of the microbial composition revealed that *Streptococcus* was the dominant genus in almost all samples, regardless of biofilm age and treatment. Thus, in vivo biofilms in this study consisted mainly of streptococci. This finding is consistent with the qPCR results. Other genera such as *Veillonella*, *Fusobacterium*, and *Gemella* were also detected in relatively high abundance; however, the bacterial composition substantially depended on the participants (Fig. 5a). Alpha diversity, which reflects the microbial community structure within individual samples, showed no significant differences among the conditions. Similarly, beta diversity, which assesses the differences in microbial composition between samples, also showed no significant differences. Specifically, for alpha diversity analysis, the Shannon diversity index, which accounts for both abundance and evenness of species, did not reveal a consistent trend across conditions (Fig. 5b). For beta diversity analysis, there was no separation by condition when looking at the dissimilarity in principal coordinates analysis (PCoA). The samples were more likely to cluster based on patients rather than conditions in the PCoA plot with the Jaccard distance matrix (Fig. 5c). That is, the variation in microbial composition was more strongly associated with individual participants than with treatment or biofilm age.

## Discussion

This study aimed to evaluate the bactericidal effects of H_2_O_2_ photolysis on in vivo dental biofilms formed on the HA specimens attached to custom-made splints. The findings confirmed that H_2_O_2_ photolysis effectively reduced bacterial viability in these biofilms; thus, the hypothesis was accepted.

The custom-made splint system with HA discs was designed to allow in vivo biofilm formation on the HA surface for ex vivo experiments, based on previous methods [22, 23]. SEM imaging confirmed the presence of biofilm on the HA surface, showing streptococci-like bacterial cells embedded in EPS. qPCR analysis from 5-day-old biofilms demonstrated that the biofilms had the same level of bacterial load in all samples subjected to analysis. While qPCR quantifies copies of the target gene, CFU counts reflect viable and culturable cells; therefore, these two assays may yield different results due to inherent differences in their detection targets. Typically, qPCR values exceed CFU counts; however, in this study, the results appeared to deviate from this trend, possibly due to limited DNA extraction efficiency [41]. Nonetheless, qPCR remains valuable for making relative comparisons across samples. Overall, both bacterial assays indicated that the biofilms randomly allocated to the test and control groups initially harbored comparable bacterial loads, thereby supporting the validity of evaluating bactericidal efficacy based on viable cell counts [10]. Microbiome analysis using NGS revealed a diverse microbial community, with *Streptococcus* as the dominant genus, a characteristic feature commonly associated with supragingival biofilms [42]. These findings confirmed that the combination approach, in which the custom-made splint and our established bactericidal assay, successfully enabled experiments using clinically relevant in vivo biofilms.

The H_2_O_2_ photolysis treatment significantly reduced the bacterial load in these complex biofilms. Specifically, the 90-s treatment was able to reduce viable bacterial counts by approximately 3 log CFU/specimen for both total bacteria and total streptococci compared with untreated controls. A previous study evaluating the bactericidal effect of H_2_O_2_ photolysis against *in vitro S. mutans* biofilms using a similar methodology reported a reduction in bacterial loads by > 4 log within 15 s at 365 nm [10]. Although the treatment duration in the present study was decided based on previous studies [10, 33], there was a difference in bactericidal efficacy between the studies. This difference likely stems from variations in biofilm composition and growth environment, as indicated previously [43]. The present study used complex and multispecies in vivo biofilms, whereas the previous study used single-species biofilms. Multispecies biofilms are generally known to exhibit greater resistance to antimicrobial treatments because of interspecies interactions, such as cooperation/competition, signaling, or DNA transfer between bacteria, resulting in enhanced adhesion and growth of biofilms or changes in EPS composition [43–45]. Furthermore, some bacteria possess catalase (i.e., catalase-positive bacteria) [46, 47], an enzyme that protects against cellular damage caused by H_2_O_2_ [48], whereas *S. mutans* lacks catalase (i.e., catalase-negative bacteria). Owing to their diverse microbial compositions, multispecies biofilms are more likely to harbor catalase-positive bacteria. NGS data from this study revealed that the biofilm of one participant exhibited a notably high abundance (approximately 15%) of *Rothia* (Micrococcaceae), a catalase-positive genus (Fig. 5a). Although *Rothia* was detected in all samples subjected to NGS, its relative abundance was lower (< 2%) in the other four participants. Additionally, *Haemophilus*, another catalase-positive genus, was detected at a low abundance in all samples. *Aggregatibacter*, which includes catalase-positive species such as *A. actinomycetemcomitans*, was also detected at low abundance, but with a high frequency (data not shown). The presence of catalase-positive bacteria could be another factor that weakened the bactericidal efficacy of H_2_O_2_ photolysis in this study, as it degrades H_2_O_2_ to water and oxygen [47, 48]. Thus, multispecies in vivo biofilms could lead to a higher catalase reaction rate than those seen in single-species biofilms formed by catalase-negative bacteria. Indeed, bubbles (i.e., oxygen) emerging from the biofilm surface were often visually observed during H_2_O_2_ immersion of the specimens (H(+)L(+) or H(+)L(−) group) in this study, possibly due to of the catalase reaction. Despite these factors, the observed 3 log reduction in bacterial load demonstrates that the H_2_O_2_ photolysis technique retains significant bactericidal activity, even against complex in vivo biofilms. Since the microbial composition of the biofilm closely resembles the actual dental biofilm as described above, this bactericidal effect may be translated to clinical applications. The qPCR results indicated that although treatment with H_2_O_2_ photolysis effectively killed bacteria in the biofilms, it did not detach or remove the biofilms from the sample surface. This suggests that the technique may best function as a supplementary method combined with some mechanical removal, rather than as a standalone therapy. Thus, this technique could be clinically used after the mechanical removal of visible dental biofilms, where bacterial loads are lower than those in intact biofilms. Notably, although the bactericidal effects observed in this clinical study design were as expected less pronounced than those observed in previous in vitro studies, they exceeded those of conventional antiseptics or aPDT, which are expected to be used as adjunctive caries treatments [10]. Thus, a 3 log reduction within 90 s against in vivo biofilms could be a promising adjunctive treatment, since conventional antiseptics and aPDT take longer time to achieve 1- or 3 log reduction, even against in vitro single-species biofilms.

Microbiome analysis revealed no significant differences in the bacterial composition in the regrowth biofilms between the groups treated with H_2_O_2_ photolysis and untreated ones. This suggests that the H_2_O_2_ photolysis treatment exerts its effect only during the treatment period and does not affect subsequent bacterial regrowth. This observation is in line with the properties of H_2_O_2_ photolysis, which generates hydroxyl radicals only during irradiation, and the hydroxyl radical is known to have a short lifetime [49]. In contrast, H_2_O_2_ photolysis applied to in vitro biofilms previously showed a post-antibiotic-like effect [50], implying the potential for compositional shifts in biofilms. Based on these findings, it was assumed that the H_2_O_2_ photolysis treatment might inhibit the regrowth of microorganisms involved in caries progression, such as acidogenic or aciduric bacteria. To assess the assumption, the splint with HA discs containing treated biofilms were worn by participants again to allow biofilm regrowth, and then the bacterial compositions or properties of 10-day-old biofilm were evaluated. However, we did not observe any distinct compositional shifts in the treated biofilms. While the bacterial composition of in vivo biofilms is strongly influenced by host factors such as diet [3], in vitro models do not account for these influences. Given that the activity of H₂O₂ photolysis under host-associated conditions remains unstudied, further investigation using in vivo biofilm is needed to elucidate its possible long-lasting or functional impacts on biofilm ecology.

This study has some limitations. First, while the participants were considered at a relatively high risk for caries, they were generally orally healthy and not necessarily caries active. The study initially aimed to establish standardized cariogenic biofilms by focusing on the presence of cariogenic bacteria rather than on existing carious lesions since this is the first study using in vivo biofilm to evaluate the effectiveness of H₂O₂ photolysis. This may have biased the biofilm composition compared with that of individuals with active caries, potentially influencing the regrowth results. Thus, it could be beneficial to evaluate biofilms obtained from volunteers who have active carious lesions. Second, the biofilm formation may have been affected by pressure from the buccal mucosa, potentially limiting bacterial attachment. While microbiome analysis showed similar bacterial profiles between 5- and 10-day biofilms, the latter may have been less mature than expected maturity [42] due to mucosal interactions refreshing the biofilm surface during the maturation period. This could partly explain the low detection rate of *S. mutans* in biofilms, despite its presence in the saliva of all the participants. In addition to the mucosal interactions, physiochemical properties (such as surface roughness) of the splint may also have affected the findings although it was not assessed in this study. Future studies should expand the participant pool to include caries-active individuals or different caries-risk profiles, characterize physiological properties of the splint, and refine the splint design to minimize interactions with oral structures, thereby better mimicking natural plaque formation.

Despite these limitations, the safety and potential clinical applicability of this technique warrant consideration. The treatment employs 3% H₂O₂, a concentration approved by the FDA for use on oral mucosa [51]. Besides, our previous animal study also demonstrated that both H₂O₂ photolysis and the application of H₂O₂ alone did not cause oral mucosal irritation [16]. Based on the demonstrated bactericidal potential and safety profile of H₂O₂ photolysis, its application for periodontal treatment has been implemented [18]. In caries treatment, the use of a rubber dam is recommended. It not only helps prevent salivary contamination but also limits unnecessary exposure of oral tissues to the treatment, which thereby enhances safety and makes this technique a safe approach for caries management. Regarding the clinical applicability, it is based on a simple concept of H₂O₂ photolysis that requires only an LED device and H₂O₂ solution, both of which are not expensive. The 90-s treatment time is likely to be clinically acceptable when applied after mechanical removal of carious dentin. As the method has a possibility to help inactivate residual biofilm in areas with limited access for mechanical approaches, it may enable greater preservation of dentin during excavation.

## Conclusion

We successfully obtained in vivo dental biofilms and provided novel evidence that H_2_O_2_ photolysis can target a diverse range of oral bacteria within in vivo dental biofilms. These findings support the potential use of H_2_O_2_ photolysis as a promising adjunctive approach for the prevention and treatment of dental caries.

## Data Availability

The data that support the findings of this study are available from the corresponding author upon reasonable request. The raw sequencing data have been deposited in the National Center for Biotechnology Information (NCBI) Sequence Read Archive (SRA) database under PRJNA1312368.

## Abbreviations

aPDT: Antimicrobial photodynamic therapy
CFU: Colony-forming unit
EPS: Extracellular polymeric substance
H₂O₂: Hydrogen peroxide
HA: Hydroxyapatite
LED: Light emitting diode
MSB: Mitis salivarius agar containing sucrose and bacitracin
NGS: Next-generation sequencing
PCoA: Principal coordinates analysis
qPCR: Quantitative real-time polymerase chain reaction
SD: Standard deviation
SEM: Scanning electron microscopy
